# Toward Environmentally Benign Electrophilic Chlorinations: From Chloroperoxidase to Bioinspired Isoporphyrins

**DOI:** 10.1021/acs.inorgchem.2c00602

**Published:** 2022-05-15

**Authors:** Silène Engbers, Ronald Hage, Johannes E. M. N. Klein

**Affiliations:** † Molecular Inorganic Chemistry, Stratingh Institute for Chemistry, Faculty of Science and Engineering, University of Groningen, Nijenborgh 4, 9747 AG Groningen , The Netherlands; ‡ Catexel BV, BioPartner Center Leiden, Galileiweg 8, Leiden 2333 BD, The Netherlands

## Abstract

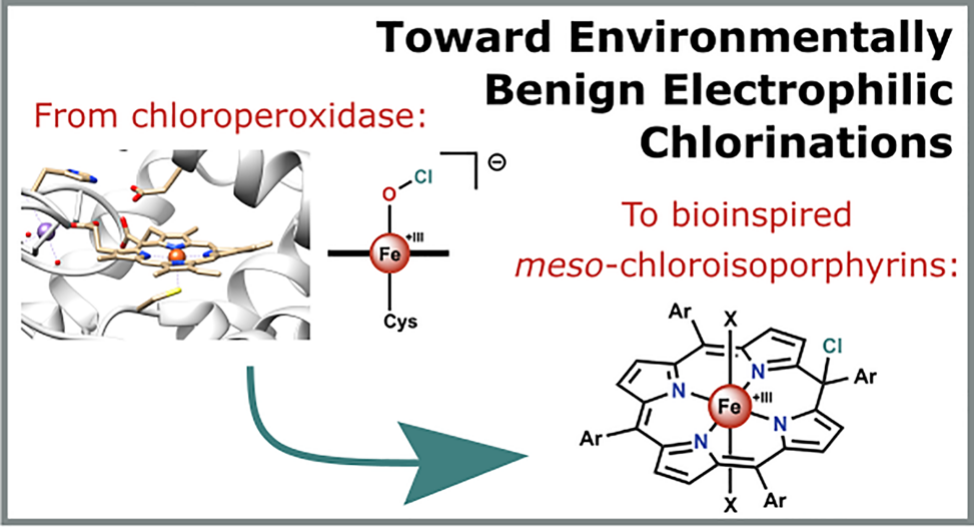

Recent desires to develop environmentally benign procedures for electrophilic chlorinations have encouraged researchers to take inspiration from nature. In particular, the enzyme chloroperoxidase (CPO), which is capable of electrophilic chlorinations through the umpolung of chloride by oxidation with hydrogen peroxide (H_2_O_2_), has received lots of attention. CPO itself is unsuitable for industrial use because of its tendency to decompose in the presence of excess H_2_O_2_. Biomimetic complexes (CPO active-site mimics) were then developed and have been shown to successfully catalyze electrophilic chlorinations but are too synthetically demanding to be economically viable. Reported efforts at generating the putative active chlorinating agent of CPO (an iron hypochlorite species) via the umpolung of chloride and using simple meso-substituted iron porphyrins were unsuccessful. Instead, a *meso*-chloroisoporphyrin intermediate was formed, which was shown to be equally capable of performing electrophilic chlorinations. The current developments toward a potential method involving this novel intermediate for environmentally benign electrophilic chlorinations are discussed. Although this novel pathway no longer follows the mechanism of CPO, it was developed from efforts to replicate its function, showing the power that drawing inspiration from nature can have.

## Abstract 2

Chloroperoxidase, first isolated in the 1960s, was found to catalyze electrophilic chlorinations via the umpolung of chloride. Fascinated by this curious transformation, efforts were made to perform such a reactivity in the absence of the enzyme, which is susceptible to hydrogen peroxide-dependent decomposition. This viewpoint describes how the field evolved into the discovery of *meso*-chloroisoporphyrins capable of electrophilic chlorinations and proposes a mechanism for a novel catalytic approach.

## Relevance of Electrophilic Chlorinations

**Figure 1:**
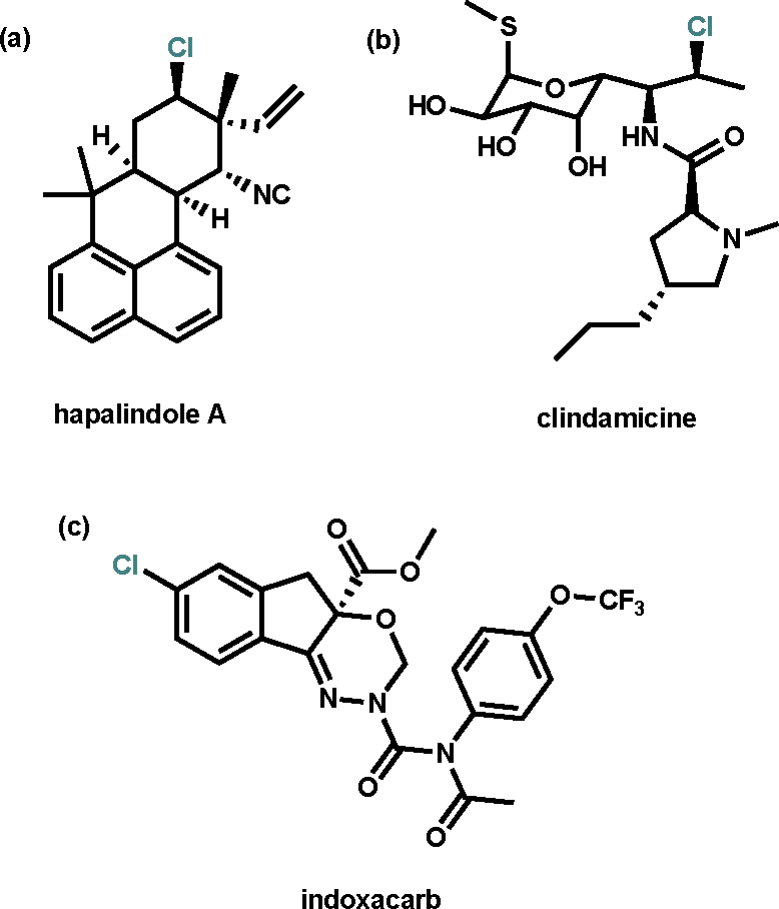
Structures of (a) a chlorinated natural product isolated from the *Stigonemataceae* family of cyanobacteria, (b) a chlorinated antibacterial drug, and (c) a chlorinated insecticide developed by DuPont.

Electrophilic chlorinations are essential chemical transformation steps as chlorinated organic compounds have an impact on many aspects of society:^1,2^ they are found in natural products (e.g., hapalindole A; Figure 1a),^3−5^ pharmaceuticals (e.g., the antibacterial drug clindamicine; Figure 1b),^6−8^ agrochemicals (e.g., the insecticide indoxacarb; Figure 1c),^9^ and organic materials.^10^ Furthermore, they are important reagents for cross-coupling reactions^11−13^ and intermediates in industrial-scale epoxidations.^14^ Nevertheless, in both industry and academia, methods for synthesizing these compounds via electrophilic chlorinations still rely on the use of chlorine gas or hypochlorite salts,^2,14−17^ which are toxic, corrosive, and nonselective. Alternatively, organic chlorinating agents, such as *N*-chlorosuccinimide or iodobenzene dichloride, are used.^18−20^ These not only require hypochlorites or chlorine gas for their synthesis^21,22^ but also generate stoichiometric amounts of organic waste upon usage. Hence, there has been a recent push to develop environmentally benign methods for electrophilic chlorinations.^23^

## Halogenations in Nature

One approach to developing a new catalytic method is to draw inspiration from nature.^24^ Halogenating enzymes can be classified into two major types: halogenases and haloperoxidases.^25−27^ Halogenases use O_2_ as their oxidant and perform halogen transfers via a radical rebound mechanism, resulting in a one-electron-oxidized halide.^25^ Haloperoxidases are hydrogen peroxide (H_2_O_2_)-dependent enzymes with the ability to perform a two-electron oxidation of a halide.^28^ Hence, only haloperoxidases perform true electrophilic halogenations.

Within the haloperoxidases, two types of metalloenzymes are most common: vanadium-dependent bromoperoxidase and heme-dependent chloroperoxidase (CPO).^25−31^ Despite its natural function being the catalysis of brominations, the former has been shown to have some chlorinating ability.^32^ Its bioinspired halogenation has been extensively studied,^33^ but only a few examples of vanadium-catalyzed chlorinations have been reported^34−38^ as the substitution of bromide for chloride has proven to be challenging.^39,40^ We will thus focus on CPO-related chlorination reactions.

## Chloroperoxidase (CPO)

**Figure 2:**
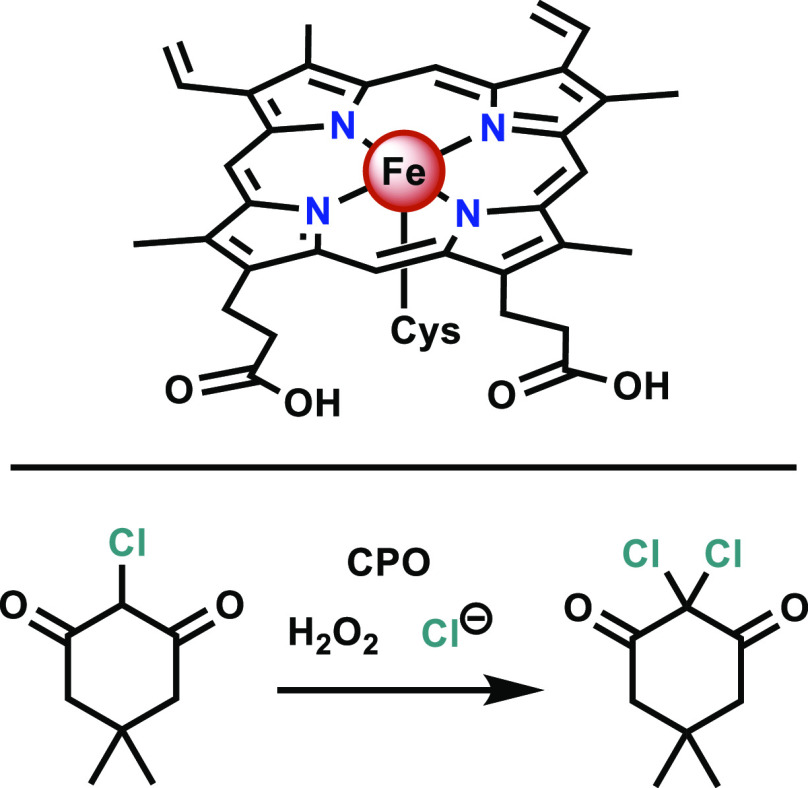
Structure of the heme complex present in the CPO active site and its model chlorination reaction with monochlorodimedone, a commonly used test substrate for CPO.^30^

In the 1930s, it was found that molds can metabolize chloride and incorporate it into organic products.^41^ In particular, the fungus *Caldariomyces fumago* was extensively studied and shown to produce caldariomycin in the presence of chloride.^41−48^ From this fungus, CPO was finally isolated and characterized in 1966,^49^ which enabled further analysis of its reactivity and mechanism.^50−70^ Structurally, CPO is a monomeric, heme-containing enzyme with a protoporphyrin IX equatorial ligand coordinating the iron (Figure 2). The active site bears polar residues on the distal side of the heme, which form a peroxide-binding site. However, unlike most peroxidases, it has a cysteine axial ligand, a feature common to cytochrome P450.^66^ Although it has been shown to perform a wide range of reaction types, including those typical of peroxidases, catalase, and P450s,^52^ its ability to catalyze electrophilic chlorinations using chloride and H_2_O_2_ at low pH values has fascinated chemists the most.^50,51,53,55,56,58,60,61,63,67^

From studies of the chlorination reactivity of isolated CPO (for an example, see Figure 2), the involvement of two main intermediates in the mechanism was inferred. H_2_O_2_ was found to react with the heme in CPO’s active site to form an iron(IV) oxo radical π-cation (Compound I),^59^ of which the structure was analyzed in detail by electron paramagnetic resonance (EPR),^59^ Mössbauer,^59^ resonance Raman,^64^ and X-ray absorption^69^ spectroscopies. Compound I can also be generated by using *m*-chloroperbenzoic acid (mCPBA).^54^ Upon reaction with chloride, a subsequent intermediate is suspected to be an Fe^III^-OCl,^53,61,63,67^ supported by Mössbauer spectral^55^ and ^35^Cl NMR studies,^56^ both of which suggest that chloride does not coordinate directly to the iron center.

## Biomimetic Complexes and Their Reactivity

**Figure 3:**
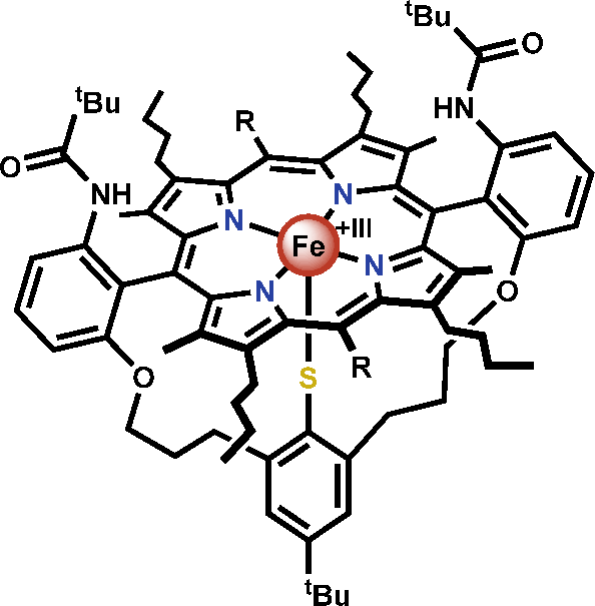
Structure of the active-site analogues developed by the Woggon group (R = H, C_6_F_5_).^71,73^

Active site analogues of CPO developed by the Woggon group (Figure 3) were designed in order to mimic both the first and second coordination spheres of CPO,^71−74^ which were elucidated in the X-ray crystal structure reported in 1995.^66^ By replicating CPO’s active site as closely as possible whilst omitting the bulk of the enzyme, it was envisioned that its mechanism of chlorination could be mimicked and further understood through the use of additional reaction assays and spectroscopic techniques.^71−74^

In addition to bearing a porphyrin ligand similar to protoporphyrin IX, the biomimetic complexes feature an axial thiolate ligand to mimic the cysteine residue present in CPO. This modification is essential because the thiolate ligand has been shown to be crucial in regulating reactivity through its redox-active nature.^62^ Furthermore, proton donors are embedded close to the free axial position, which simulates a glutamate residue in the active site of CPO that is conveniently positioned to protonate intermediates in the reaction.^66,72^

With the help of these complexes, the chlorination mechanism was further investigated.^71,72^ The same species was generated when the iron(III) complex was reacted with either H_2_O_2_ and subsequently chloride or a hypochlorite source, further supporting the generation of an Fe^III^-OCl intermediate.^71,72^ In addition, it was found that the Fe-OCl intermediate is likely protonated prior to substrate chlorination, an aspect that is of relevance to the subsequent step involving chlorination of the substrate.^71,72^ Moreover, these complexes were shown to catalytically chlorinate monochlorodimedone as well as other cyclic ketones and aromatic compounds.^73^

**Scheme 1:**
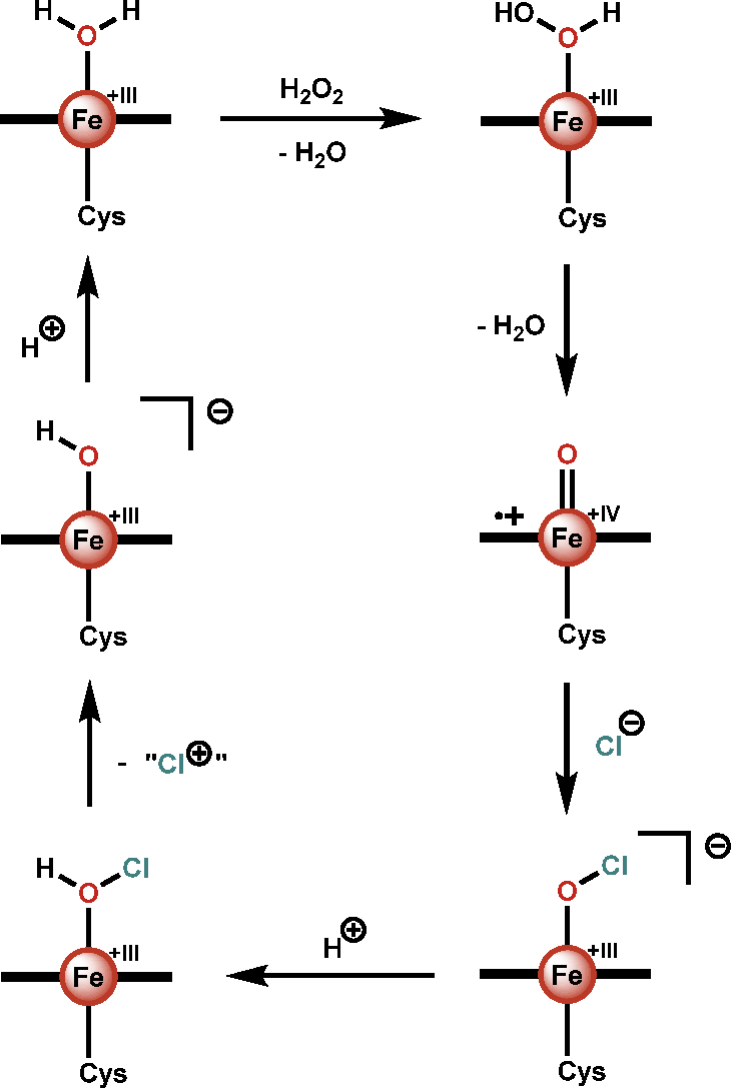
Proposed Mechanism of Chloride Oxidation by CPO The equatorial ligand, here schematically depicted as a horizontal bold line, is protoporphyrin IX.

In combination with the data obtained from studying CPO, the biomimetic complexes from the Woggon group enabled the mechanism to be further understood (Scheme 1).^72^ The resting state is thought to be an iron(III) species that is oxidized by H_2_O_2_ to Compound I. This can then react with a chloride to form the putative Fe^III^-OCl species, which is likely protonated before chlorination of the substrate occurs. It should be noted that while some studies suggest that substrate chlorination occurs directly from the (protonated) enzyme-bound Fe-OCl adduct,^61,63,72^ others have argued that, because X-ray crystallography reveals an enzyme active site lacking a substrate-binding pocket,^68^ HOCl must be released into the solution prior to substrate chlorination.^75^ In either case, a two-electron oxidation of chloride is achieved, thereby allowing the transfer of a putative “Cl^+^” species to a desired substrate.^51^

## Toward Industrial Relevance: Bioinspired Developments

Although CPO has been shown to chlorinate a wide variety of substrates, including those featuring alkene, alkyne, and aryl groups,^30,76,77^ the large-scale industrial application of CPO as a catalyst for electrophilic chlorinations appears challenging. Not only do most relevant substrates have low solubility in water, but CPO, like most peroxidases, suffers from peroxide-dependent inactivation.^78^ Efforts have been made to find ways around these challenges.^78−81^ However, significant developments are still required before CPO becomes industrially relevant as a catalyst for electrophilic chlorinations. The Woggon complexes have, of course, been shown to be suitable CPO mimics and are capable of performing electrophilic chlorinations catalytically, with turnover numbers ranging from 10 to 1500 in the presence of Lewis acids (for example, ZnCl_2_).^71,73,74^ Unfortunately, their syntheses are also lengthy and thus unlikely to be suitable for industrial usage.^71,73^ The use of a simpler ligand framework, however, may yield a fruitful method for electrophilic chlorinations.

**Scheme 2:**
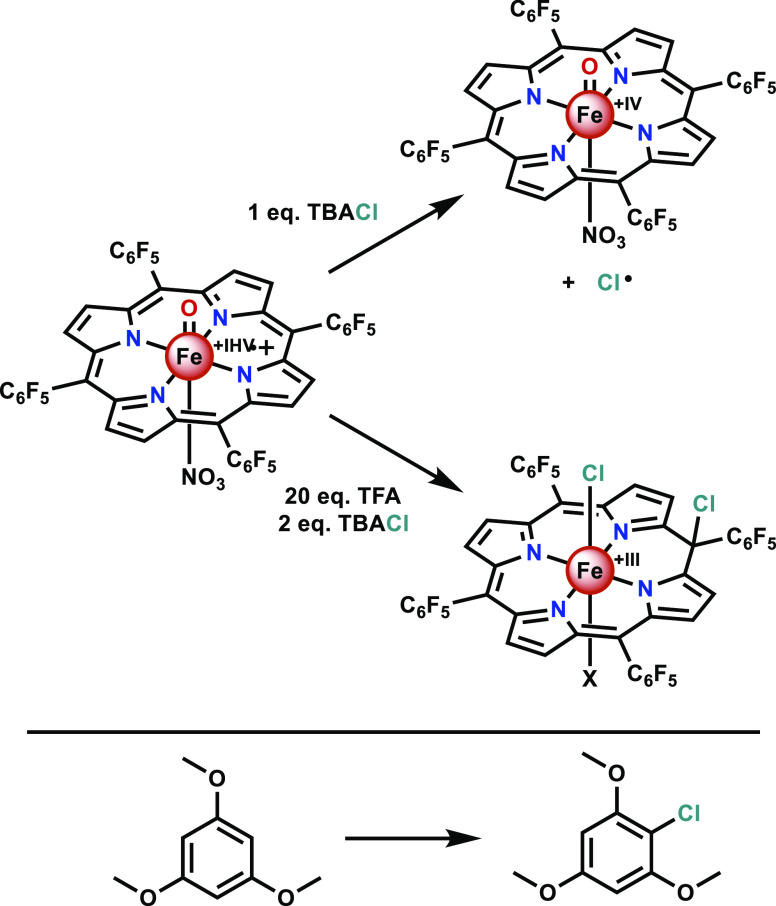
Reactivity of Compound I with Tetrabutylammonium Chloride (TBACl) in the Absence (top) and Presence (bottom) of TFA, Studied by the Fujii Group^82,84^ as well as their Chlorination Reactivity of 1,3,5-Trimethoxybenzene Reactions are performed in DCM at −90 °C. X depicts either trifluoroacetate or NO_3_^–^ as the axial ligand.

The Fujii group employed simple meso-substituted iron porphyrin complexes in attempts to mimic the chlorination reactivity of CPO.^82−84^ Compound I was initially generated by the reaction of (TPFPP)Fe-NO_3_, where TPFPP = *meso*-tetra(pentafluorophenyl)porphyrin, with ozone in dichloromethane (DCM) at −90 °C. The subsequent addition of chloride to the newly formed Compound I led to a one-electron transfer from chloride to the iron complex, yielding back an iron(IV) oxo species (Compound II) and a chloride radical (Scheme 2, top).^82^ Hence, only a one-electron oxidation of chloride occurred rather than the expected two-electron oxidation. Complexes bearing more electron-donating meso substituents were later screened, and none were found to form the desired iron hypochlorite either.^83^

Interestingly, when chloride was added to (TPFPP^+**·**^)(NO_3_)Fe=O in the presence of excess trifluoroacetic acid (TFA) in DCM at −90 °C, a two-electron oxidation was observed. However, rather than an iron hypochlorite being formed, the active chlorinating species in CPO, a *meso*-chloroisoporphyrin, was generated (Scheme 2, bottom). This species was observed by UV–vis spectroscopy and further characterized by NMR, EPR, and electrospray ionization mass spectrometry.^84^ Recently, the crystal structure of a similar *meso*-chloroisoporphyrin was published by the McDonald group.^85^ Upon reaction with cyclohexene, 1,3,5-trimethoxybenzene, and anisole, the isoporphyrin was shown to be capable of electrophilic chlorinations (Scheme 2), yielding back an iron(III) porphyrin complex in the process.^84^ Thus, although these simple complexes show reactivity that largely deviates from that of CPO and the biomimetic complexes from the Woggon group, they can form transient species that bear promise as electrophilic chlorinating agents.

**Scheme 3:**
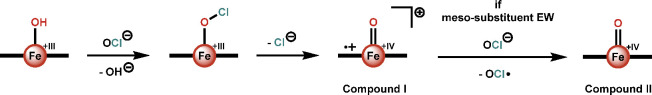
Mechanism of (porphyrin)Fe-OCl Formation and Decomposition^,^^83^ EW = electron withdrawing. The equatorial ligand, here schematically depicted as a horizontal bold line, is a meso-substituted porphyrin.

It should be noted that (TPFPP)Fe-OCl can be generated from (TPFPP)Fe-OH by ligand exchange with tetrabutylammonium hypochlorite (TBAOCl) in DCM/acetonitrile (1:1) at −60 °C.^86^ At room temperature though, this species rapidly decomposes to Compound II. At first glance, this would indicate that the FeO–Cl bond has a tendency to cleave homolytically, generating only a one-electron-oxidized chlorine species. However, upon further inspection and screening of complexes with more electron-donating meso substituents, it appeared that the FeO–Cl bond breaks heterolytically to form Compound I. In the presence of excess TBAOCl, complexes for which Compound I has a reduction potential larger than that of the hypochlorite anion are reduced from Compound I to Compound II by an electron-transfer process. This is true for complexes bearing strongly electron-withdrawing meso substituents, such as 2,6-dichlorophenyl and 2,4,6-trichlorophenyl (Scheme 3).^83^ The fact that heterolytic cleavage of the O–Cl bond occurs, forming Compound I and chloride, indicates that, by microscopic reversibility, biomimetic formation of an Fe-OCl should be possible. It might just not be energetically accessible.

Similar to *meso*-chloroisoporphyrin, (TPFPP)Fe-OCl is capable of chlorinating 1,3,5-trimethoxybenzene (Scheme 2). However, it epoxidizes cyclohexene rather than chlorinating it, as shown for the isoporphyrin.^86^ Hence, the isoporphyrin not only is more accessible than the iron porphyrin hypochlorite but appears to be a superior chlorinating agent. The challenge remains to transform the stoichiometric chlorination, which employs a preformed *meso*-chloroisoporphyrin, into a functioning catalytic pathway. For this, a further understanding of the possible mechanism is required.

**Scheme 4:**
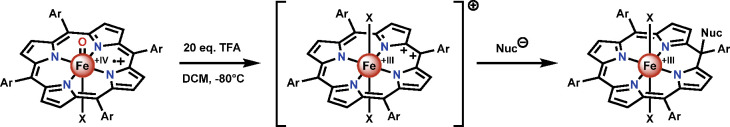
Identification of the π-Dication Intermediate in the Pathway toward an Isoporphyrin^,^^92^ Ar = 2,6-difluorophenyl. Nuc = a nucleophile (e.g., 4,5-dimethylimidazole or chloride). X represents any anionic coordinating ligand present in solution.

The generation of Compound I for meso-substituted iron porphyrin complexes is relatively well understood.^87−91^ On the contrary, the conversion of Compound I to an isoporphyrin under acidic conditions remains largely understudied. However, a recent study by the Karlin group does elucidate one of the intermediates. When (TDFPP)Fe(SbF_6_), where TDFPP = 5,10,15,20-tetrakis(2,6-difluorophenyl)porphyrin, was oxidized to Compound I and subsequently reacted with TFA, a transient iron(III) π-dication species was formed. This was then shown to react with nucleophiles (including chloride) to generate an isoporphyrin (Scheme 4).^92^ Hence, we expect the formation of a *meso*-chloroisoporphyrin to proceed through an iron(III) complex bearing a doubly oxidized porphyrin ligand.

These iron(III) π-dication species, first characterized in 1993 by UV–vis, ^1^H NMR, and EPR,^93^ are rather uncommon, and only a few examples of π-dication metalloporphyrin complexes have been reported in general.^94−96^ Interestingly, however, it was already proposed in 1970 that isoporphyrins could be synthesized by an electrochemical 2-fold oxidation of a zinc(II) species in the presence of a nucleophile.^97^

## Potential Bioinspired Catalytic Approach

**Scheme 5:**
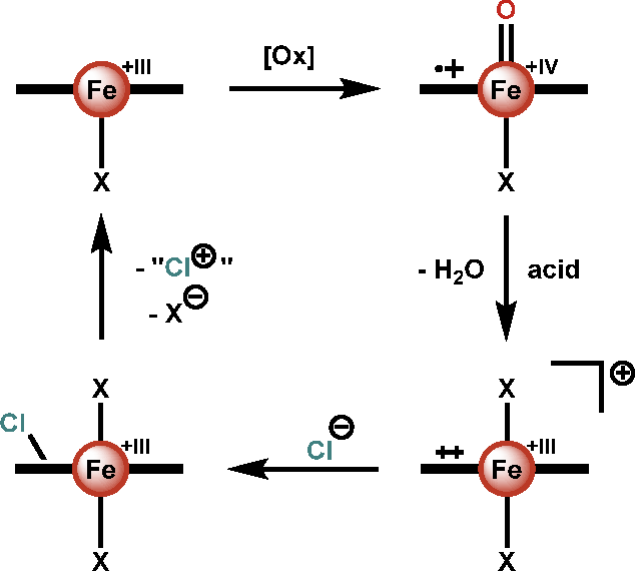
Possible Catalytic Cycle for Bioinspired Iron-Catalyzed Electrophilic Chlorinations Employing Simple Iron Porphyrin Species [Ox] refers to any oxidant capable of oxidizing to Compound I. X represents any anionic coordinating ligand in solution. The equatorial ligand, here schematically depicted as a horizontal bold line, is a meso-substituted porphyrin.

From the aforementioned data available in the literature, we can infer a potential catalytic cycle for bioinspired iron-catalyzed electrophilic chlorinations employing simple iron porphyrin complexes (Scheme 5). An iron(III) species is first oxidized to Compound I. Commonly, this is done through the use of mCPBA or ozone.^90^ However, the use of the more environmentally benign oxidant H_2_O_2_ is possible under certain conditions.^87,88^ As was elucidated by the Karlin group, Compound I converts to a π-dication by reaction with acid.^92^ Chloride can then attack the porphyrin ring, forming a *meso*-chloroisoporphyrin, which is capable of electrophilic chlorinations. Upon chlorination from an isoporphyrin, an iron(III) species is recovered,^84^ allowing the catalytic cycle to be closed. Although each of the transformations has been performed sequentially, no attempts at catalysis have been reported. Thus, we emphasize that the proposed catalytic cycle is for now purely hypothetical and has yet to be shown experimentally viable. We are hopeful though that, with further research, it could lead to a novel method for environmentally benign electrophilic chlorinations.

## Conclusion

Although the proposed direction outlined here has deviated largely from the mechanism of CPO, we believe that the hypothetical bioinspired approach, composed from several experimental observations, holds promise. In contrast to the biomimetic route, the proposed alternative does not form (toxic and corrosive) hypochlorites at any point in the cycle, possibly making it even more environmentally benign. Moreover, the *meso*-chloroisoporphyrins could be more specific because they are unlikely to yield epoxidation products instead of the desired chlorinations, which has been reported to occur for iron hypochlorite species.^86^ Furthermore, we hope to have demonstrated how drawing inspiration from nature can be a powerful tool and open up novel avenues in the pursuit of exploring chemical reactivity.
